# Bibliometric analysis of orexin: A promising neuropeptide

**DOI:** 10.1097/MD.0000000000040213

**Published:** 2024-10-25

**Authors:** Ning Sun, Rui Wei, Bochao Jia, Taiwei Lou, Zirong Li, Xiaowei Nie, Wenxiao Yu, Ming Zhao, Qiuyan Li

**Affiliations:** a Xiyuan Hospital of China Academy of Chinese Medical Sciences, Beijing, China; b Post-Doctoral Research Station, Xiyuan Hospital of China Academy of Chinese Medical Sciences, Beijing, China; c Guang’anmen Hospital, China Academy of Chinese Medical Sciences, Beijing, China; d Beijing University of Chinese Medicine, Third Affiliated Hospital, Beijing, China; e Department of Andrology, Xiyuan Hospital, China Academy of Chinese Medical Sciences, Beijing, China; f Department of Andrology, Wang Jing Hospital, China Academy of Chinese Medical Sciences, Beijing, China.

**Keywords:** mood disorders, neurodegenerative diseases, neurology, orexin, sleep initiation and maintenance disorders

## Abstract

**Background:**

Orexin is an excitatory neuropeptide produced in the lateral hypothalamus, playing a role in various physiological functions in humans. There is a growing body of literature on orexins. This paper utilizes CiteSpace software to organize and analyze a significant number of articles on orexin, providing readers with an intuitive overview of research trends and emerging hot topics in this field.

**Methods:**

The electronic database, Web of Science Core Collection (WoSCC), was searched for publications related to orexins. Annual publications, countries/regions, institutions, authors and keywords were analyzed, and the results were visualized via CiteSpace software.

**Results:**

A total of 5486 publications were included, with articles making up 85.30% and reviews 14.70%. The top 3 countries publishing the most papers on orexins were the United States (2057 papers), Japan (778), and China (556). The leading institutions included Research Libraries UK (278), Harvard University (250), and Stanford University (221). The most prolific authors in the field were Yves Dauvilliers (69), Abbas Haghparast (67), and Takeshi Sakurai (66). The most frequently used keywords were “neurons” (981), followed by “sleep” (824), “food intake” (612), “receptors” (547), and “neuropathology” (535). Recent research hotspots include melanin-concentrating hormone neurons, Alzheimer disease, gamma-aminobutyric acid neurons, oxidative stress, suvorexant, the orexin system, prevalence, and stress. Based on keyword clustering analysis, the top 5 research hotspots from 2003 to 2022 were: the effects of orexins on sleep and metabolism, potential pathways of orexin signaling, the relationship between orexin and immunity, new findings on depression and hypertension related to orexin, and possible targets for neurodegenerative diseases.

**Conclusion:**

Orexin, a neuropeptide linked to various physiological and pathological processes, plays a crucial role in sleep/wakefulness, reward mechanisms, stress responses, and neurodegenerative diseases. Its significant research value and potential medical applications are underscored by the rapid expansion of studies, particularly in the USA and Japan. However, the lack of collaboration among researchers highlights the need for enhanced academic exchange and cooperation to further advance the field of orexin research.

## 1. Introduction

Orexin, also known as hypocretin, is an excitatory neuropeptide discovered in 1998 by 2 independent research teams led by De Lecea and Sakurai. The orexin system comprises orexin A, orexin B, and their corresponding receptors: orexin type 1 receptor and orexin type 2 receptor. This system plays a crucial role in various physiological processes, including the regulation of the sleep/wake cycle, motivation, energy homeostasis, and emotional responses.^[1–3]^ In the past 2 decades, the number of articles on orexin has surged significantly,^[4]^ reflecting the growing interest in orexin. Notably, a major milestone was reached when the efficacy of orexin receptor antagonists for treating insomnia was definitively established, leading to their approval for clinical use.^[5–8]^

Orexin is recognized as a crucial regulator of sleep and wakefulness, playing a pivotal role in insomnia and sleep disorders. In recent years, a significant breakthrough has been the approval of dual orexin receptor antagonists, such as suvorexant, lemborexant, and daridorexant, as marketed treatments for insomnia. Unlike traditional sleep medications, such as benzodiazepines and “Z-drugs” (non-benzodiazepines that begin with the letter “Z”), which have been used for decades, the new orexin receptor antagonists do not modulate the activity of γ-aminobutyric acid receptors—the primary inhibitory mechanism in the central nervous system. Instead, these antagonists temporarily block the orexin pathway, resulting in distinct effects, including reduced morning or next-day sedation, decreased motor coordination, and less cognitive impairment. On the other hand, evidence suggests that narcolepsy is associated with a decrease in orexin levels in the brain and cerebrospinal fluid. Currently, a phase II clinical trial is underway for an oral orexin receptor agonist.^[9,10]^

Orexins play a vital role in modulating stress-related behaviors and emotions by enhancing the activity of the hypothalamic–pituitary–adrenal axis and the sympathetic nervous system. The selective orexin receptor 2 antagonist seltorexant has shown promise in treating depression; However, further clinical trials are needed to establish its efficacy.^[11–13]^

Orexin 1 receptors play pivotal roles in motivational behavior and reward mechanisms, closely linked to addiction to alcohol and cocaine. Increased expression of the orexin system can transform physiological needs or exposure to rewarding opportunities into organized, goal-directed behaviors. In addicted states, environmental cues signaling drug availability strongly activate orexinergic neurons, triggering downstream circuits that promote drug-seeking behaviors. Although the plasticity mechanisms of the orexin system remain unclear, it is viewed as a promising therapeutic target for substance use disorders, including alcohol and cocaine addiction.^[14]^

In 2023, a subnetwork of the orexin system in the human hippocampus linked to weight and diet was discovered in *Nature*, presenting a promising target for addressing obesity and eating disorders, such as Prader–Willi syndrome. This subnetwork circuitry originates from the hypothalamus and extends to the dorsolateral aspect of the hippocampus. Using techniques like combined tractography, intracranial electrophysiology, cortico-subcortical evoked potentials, and brain-clearing three-dimensional histology, structural and functional analyses were conducted in a human cohort with dysregulated eating behavior. The findings revealed a connectivity pattern that inversely correlated with body mass index. Additionally, preliminary research on the relationship between orexin and blood pressure, as well as cognitive functions, suggests numerous potential avenues for further exploration.^[15]^

Given the extensive body of research on orexin and its diverse physiological functions, gaining a comprehensive understanding of its research landscape within a limited timeframe is challenging. Therefore, we have employed bibliometric methods to visually represent the current research status and highlight key areas of interest in the field of orexin for our readers.

## 2. Materials and methods

### 2.1. Data source

Our data was extracted from the WoSCC, including journals, titles, abstracts, keywords, author information, and cited references, all downloaded in TXT format. To avoid bias resulting from frequent updates to the WoSCC database, data were retrieved and downloaded on the same day (June 1, 2023). Two authors (N.S. and W.X.Y.) independently conducted the systematic literature search and achieved highly consistent results (99% similarity). In the event of a dispute, the senior author Q.Y.L. will serve as the arbitrator.

A total of 6917 published papers were identified based on specific criteria, including the topic (“orexin*” OR “hypocretin*”), the language (English), and a timeframe from 2003 to 2022. By refining the types of publications, we excluded editorial materials, news items, letters, and proceeding papers etc, retaining only articles and reviews, resulting in a total of 5677 publications. We then removed duplicates, incomplete, or unrelated papers, resulting in a final total of 5486 publications. Our data were entirely sourced from the database; therefore, approval from the Ethics Committee was not required.

### 2.2. Statistical analysis

To provide comprehensive and objective results, various characteristics of the included publications were analyzed both qualitatively and quantitatively in our study. The K values were established at 5 and 10 for the g-index in the analysis of authors and keywords, respectively, serving as criteria for selection. Then, the number of papers published by each author and the frequency of keywords were sorted in descending order to identify the top authors and keywords.

The bibliometric analysis was conducted using CiteSpace (version 6.2.R2) to visualize networks of countries or regions, institutions, authors, and keywords. In the maps generated by CiteSpace, different elements such as countries/regions, institutions, authors, and keywords are represented by distinct nodes. The size of the node represents frequency, with larger nodes indicating higher frequencies of the element. The line between different nodes represents their relationships. A darker color indicates a higher frequency of co-occurrence between 2 nodes, signifying a stronger association between them.

The parameters for CiteSpace were set as follows: time slicing (2003–2022), with 1 year per slice; term source (all selected); selection criteria (g-index *k* = 5–15). For network pruning, the Pathfinder and slicing network pruning algorithms were chosen. Clustering tags for keywords were generated using the Latent Semantic Indexing (LSI) modularity values algorithm. LSI is one of the 3 common algorithms for extracting cluster label terms in CiteSpace, alongside log-likelihood rate and mutual information. A higher LSI value indicates better representativeness of the cluster; Visualization (cluster view static and display merged network).

## 3. Results

### 3.1. Distribution of annual publications

**Figure 1.**
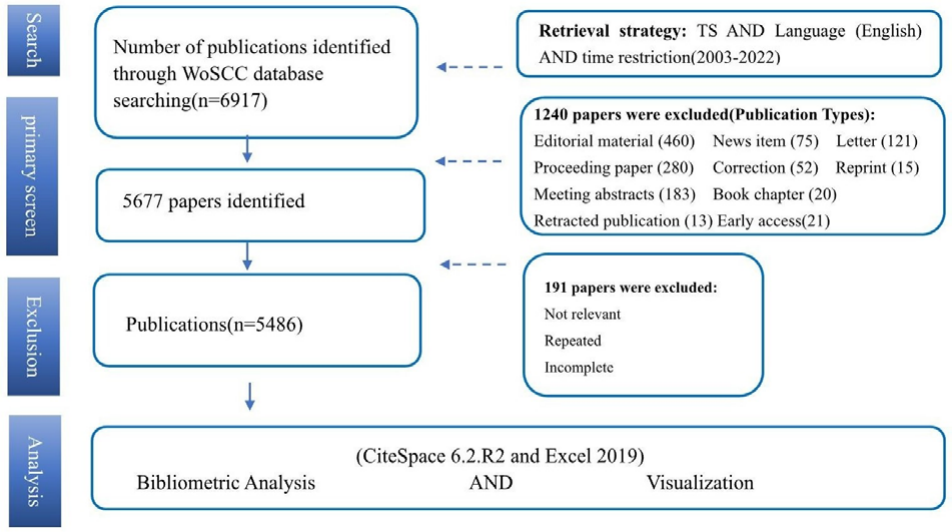
Research Flowchart. TS = topic search, WoSCC = Web of Science Core Collection.

**Figure 2.**
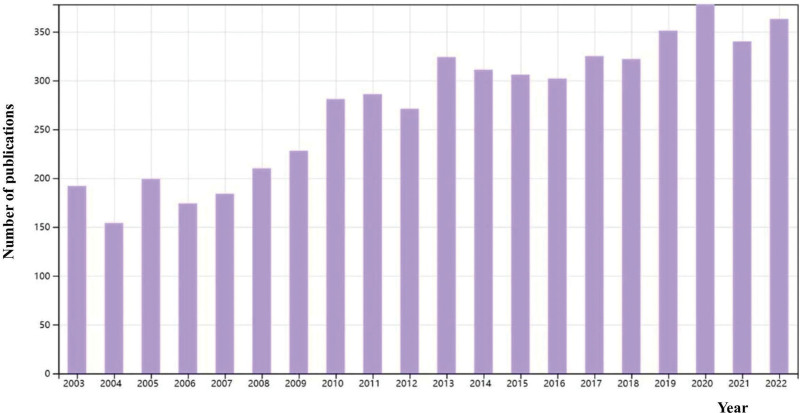
The growth trend of the publications on orexin from 2003 to 2022.

**Figure 3.**
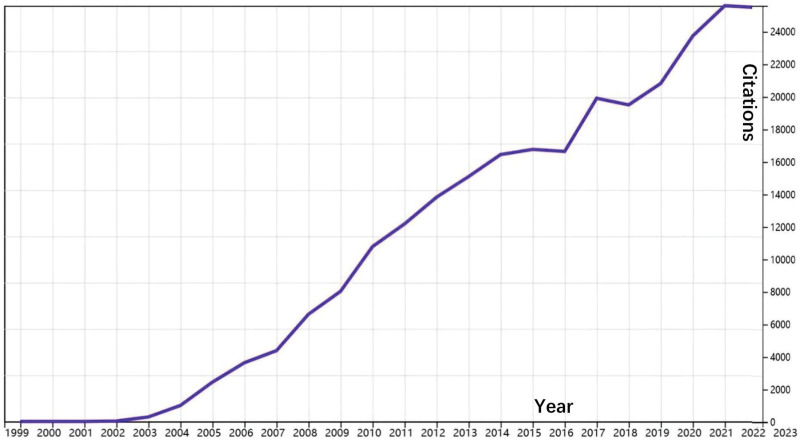
Number of citations of the publications on orexin from 2003 to 2022.

A total of 5486 publications remained, consisting of 85.30% articles and 14.70% reviews. Figure 1 illustrates the workflow of our study. There were 350 papers published in 2022. Figure 2 shows the growth trend of annual publications on orexins, which increased from 192 in 2003 to 363 in 2020. According to the WoSCC database, the 5486 papers received a total of 207,699 citations, with an average of 37.76 citations per paper (Fig. 3).

### 3.2. Analysis of countries or regions

**Figure 4.**
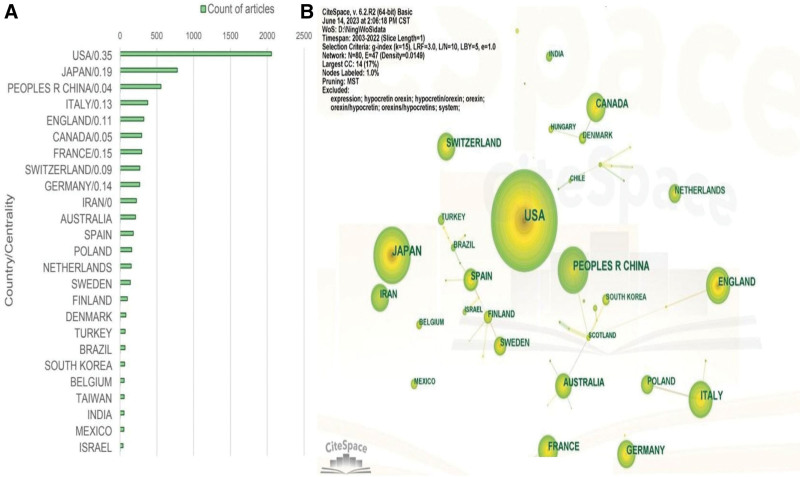
(A) The top 25 most productive countries or regions. USA indicates the United States; and People’s R China, People’s Republic of China. (B) The cooperation network of countries or regions in the field of orexin research.

From 2003 to 2022, 80 countries or regions contributed to research publications on orexin. The global geographic distribution of these publications was analyzed, and the most productive countries or regions were identified based on data from the WoSCC. Figure 4A displays the top 25 most productive countries or regions in orexin research. The United States was the most productive country, with 2057 papers published, followed by Japan (778), China (556), Italy (376), England (322), Canada (295), France (295), Switzerland (269), Germany (267), and Iran (222). The centrality values for the USA and Japan are significantly >0.1, indicating their prominent roles within the network. In contrast, the betweenness centrality values for China and Iran are below 0.05, suggesting their independent position within the network.

### 3.3. Analysis of institutions

**Figure 5.**
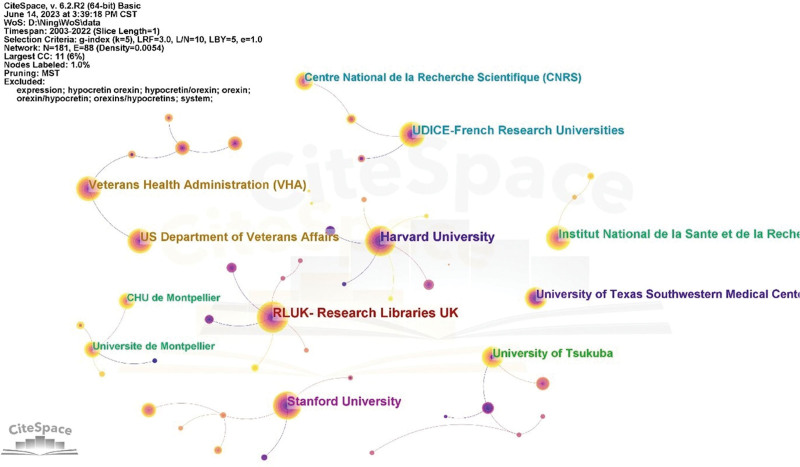
The cooperation network of institutions in the field of orexin. CHU = Centre Hospitalier Universitaire, RLUK = Research Libraries of the United Kingdom.

As shown in Table 1, the Research Libraries of the United Kingdom (RLUK) was the most productive institution, with 278 papers published, followed by the Harvard University (n = 250), Stanford University (n = 221), Institution national de la santé et de la recherche médicale (n = 190), United States Department of Veterans Affairs (n = 186), University of California System (n = 186), Veterans Health Administration (VHA) (n = 186), UDICE-French Research Universities (n = 173), University of Texas Southwestern Medical Center Dallas (n = 136), and University of Tsukuba (n = 132) successively. The institutional collaboration network was mapped using CiteSpace, with minimum thresholds of 50 papers published and 500 citations. As shown in Figure 5, 22 out of the 181 institutions were identified. The results indicate that the centrality values for RLUK, Stanford University and University of Tsukuba exceed 0.1, suggesting that they have more connections with other nodes in the network. In contrast, the centrality values for Institution national de la santé et de la recherche médicale, University of California System and University of Texas Southwestern Medical Center Dallas are below 0.1, suggesting that they have a relatively independent position within the network.

**Table 1:** The top 10 most productive institutions in the field of orexin research.

Rank	Institutions	Counts	Centrality
1	RLUK- Research Libraries UK	278	0.4
2	Harvard University	250	0.11
3	Stanford University	221	0.19
4	Inserm	190	0.07
5	US Department of Veterans Affairs	186	0.12
6	University of California System	186	0.08
7	Veterans Health Administration (VHA)	186	0.1
8	UDICE-French Research Universities	173	0.12
9	University of Texas Southwestern Medical Center Dallas	136	0.04
10	University of Tsukuba	132	0.28

Inserm = Institut National de la Santé et de la Recherche Médicale, RLUK = Research Libraries of the United Kingdom.

### 3.4. Analysis of authors

**Figure 6.**
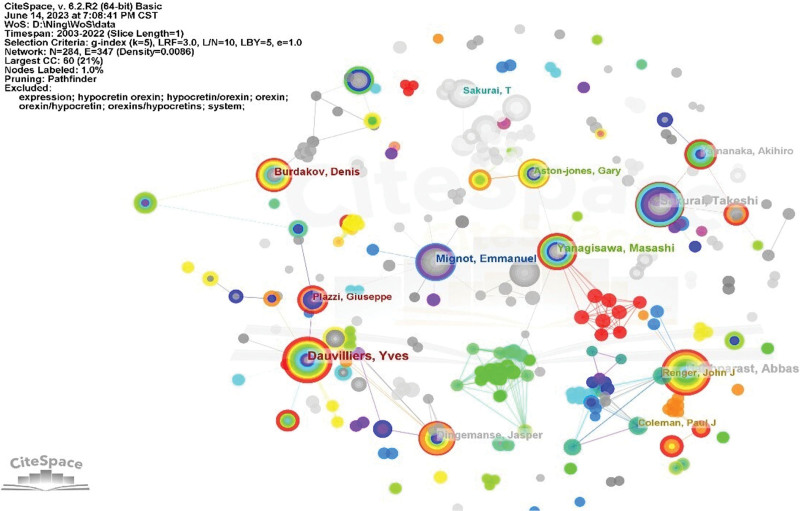
Co-authorship network in the field of orexin research.

The top 10 most productive authors in the field of orexin research are listed in Table 2. Among them, Yves Dauvilliers (orcid ID: 0000-0003-0683-6506) ranked first, with 69 papers published over the last 20 years, followed by Abbas Haghparast (orcid ID: 0000-0003-1084-180X) (n = 67), and Takeshi Sakurai (orcid ID: 0000-0002-7989-8882) (n = 66). Emmanuel Mignot (orcid ID: 0000-0002-6928-5310) (n = 46), Masashi Yanagisawa (orcid ID: 0000-0002-7358-4022) (n = 45), Jasper Dingemanse (orcid ID: 0000-0002-4083-5817) (n = 36) Denis Burdakov (orcid ID: 0000-0002-9134-9165) (n = 33), Akihiro Yamanaka (orcid ID: 0000-0001-6099-7306) (n = 29), Paul J Coleman (orcid ID: 0000-0001-8094-8341)(n = 28), and Gary Aston-jones(orcid ID: 0000-0002-5034-3816) (n = 27). Thus, these authors can be recognized as leaders in the field of orexin research. CiteSpace software was utilized to create the co-authorship overlay visualization network, requiring authors to have a minimum of 20 published papers. Studies published from 2003 to 2022 were selected for analysis, with a time slice of 1 year. The co-authorship network is displayed in Figure 6. Each cluster typically comprises multiple authors. The size of each circle in the diagram corresponds to the number of studies published by the author, while the thickness of the line connecting 2 circles indicates the frequency of co-authorship between researchers. The number of lines linking the circles provides insights into the extent of their collaboration. Circles of the same color denote authors belonging to the same cluster. Specifically, blue nodes represent earlier published studies, while yellow nodes signify more recent studies. This visualization reveals the prevalence of established partnerships among authors and the presence of distinct author clusters in the collaborative network. Centrality, as shown in Table 2, is a concept used to measure how close a node is to the center of the network; a higher centrality value indicates greater importance of the node. If the centrality value exceeds 0.1, the author is considered to have a greater impact. The centrality values for all authors are below 0.1, indicating their relatively limited influence within the network.

**Table 2:** The top 10 most productive authors in the field of orexin research.

Rank	Author	Article counts	Centrality	Year
1	Dauvilliers, Yves	69	0.05	2010
2	Haghparast, Abbas	67	0	2013
3	Sakurai, Takeshi	66	0.02	2006
4	Mignot, Emmanuel	46	0.02	2006
5	Yanagisawa, Masashi	45	0.05	2006
6	Dingemanse, Jasper	36	0	2013
7	Burdakov, Denis	33	0.01	2007
8	Yamanaka, Akihiro	29	0	2013
9	Coleman, Paul J	28	0	2010
10	Aston-jones, Gary	27	0.01	2009

### 3.5. Analysis of keywords

**Figure 7.**
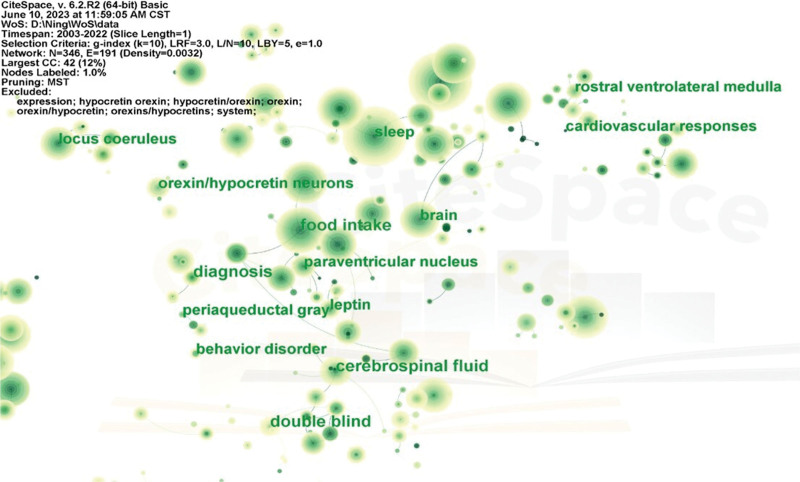
Analysis of co-occurring keywords in research papers on orexins.

For our study, we selected publications from 2003 to 2022 for the CiteSpace analysis, using a time slice of 1 year. The highest frequency keywords were identified from the included publications, with the top 31 keywords (appearing more than 200 times) presented in Table 3. The co-occurring keywords shown in Figure 7 highlight the prominent topics within the field of orexin research. Nodes represent keywords, and the size of each node corresponds to the frequency of co-occurrence. The keyword with the highest frequency was “neurons” (981), followed by “sleep,” “food intake,” “receptors,” and “neuropathology.” The majority of nodes are represented by dark green circles, indicating their significance through strong betweenness centrality. These nodes exhibit the most pronounced citation bursts and signify emerging trends in orexin research.

**Table 3:** High-frequency keywords in the field of orexin research.

Rank	Keywords	Frequency	%	Year
1	Neurons	981	2.03	2003
2	Sleep	824	1.71	2003
3	Food intake	612	1.27	2003
4	Receptors	547	1.13	2003
5	Orexin A	535	1.11	2003
6	Narcolepsy	517	1.07	2003
7	Rat	502	1.04	2003
8	Melanin concentrating hormone	496	1.03	2003
9	Lateral hypothalamus	438	0.91	2003
10	Brain	414	0.86	2003
11	Neuropeptide Y	412	0.85	2003
12	Orexin neurons	393	0.81	2003
13	Messenger RNA	393	0.81	2003
14	Ventral tegmental area	381	0.79	2004
15	Activation	378	0.78	2003
16	Rat brain	365	0.76	2003
17	Gene expression	350	0.72	2003
18	Locus coeruleus	340	0.70	2003
19	REM sleep	312	0.65	2003
20	Nucleus accumbens	305	0.63	2005
21	Hypothalamus	282	0.58	2003
22	Peptides	281	0.58	2003
23	Mice	267	0.55	2003
24	Cerebrospinal fluid	250	0.52	2003
25	Energy homeostasis	246	0.51	2003
26	Central nervous system	225	0.47	2003
27	Wakefulness	223	0.46	2003
28	Cataplexy	217	0.45	2003
29	Feeding behavior	213	0.44	2003
30	Corticotropin releasing factor	206	0.43	2003
31	Body weight	205	0.42	2003

**Figure 8.**
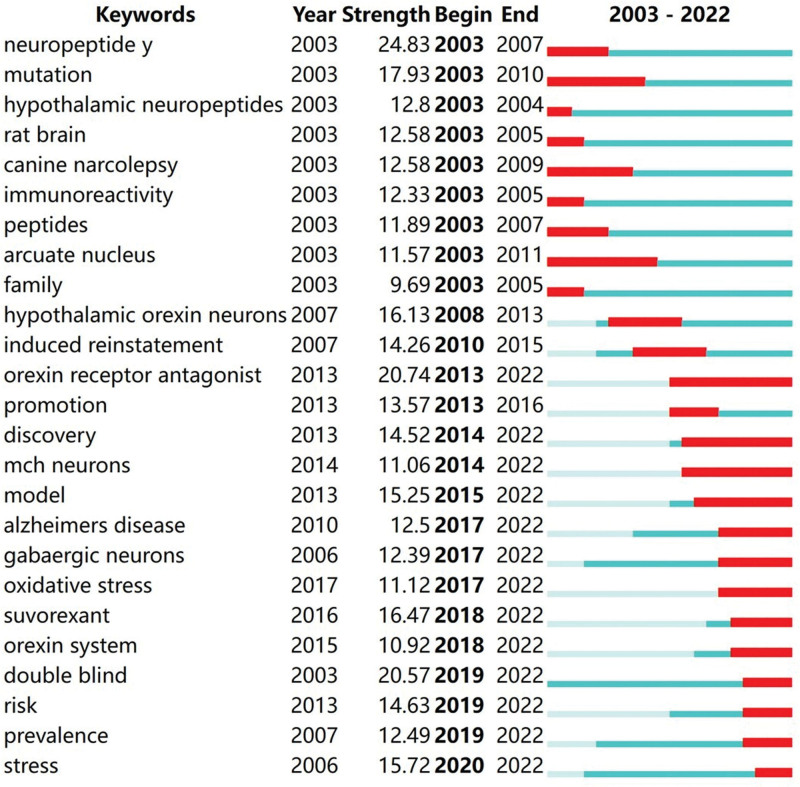
Keywords with the strongest citation bursts. (Keywords marked in red indicate a sudden increase in the usage frequency during that period. Blue represents a relatively unpopular time period.)

The top 25 keywords exhibiting the most significant citation bursts were arranged in chronological order. Figure 8 illustrates the history of orexin research. The red lines indicate the periods of the strongest citation bursts for keywords, while blue lines represent less popular periods. Recent research hotspots include orexin receptor antagonists, discovery, melanin-concentrating hormone neurons, models, Alzheimer disease, GABAergic neurons, oxidative stress, suvorexant, the orexin system, double-blind studies, risk, prevalence, and stress. Moreover, the red lines for these keywords show no signs of attenuation towards blue, suggesting that they are likely to remain popular moving forward.

**Figure 9.**
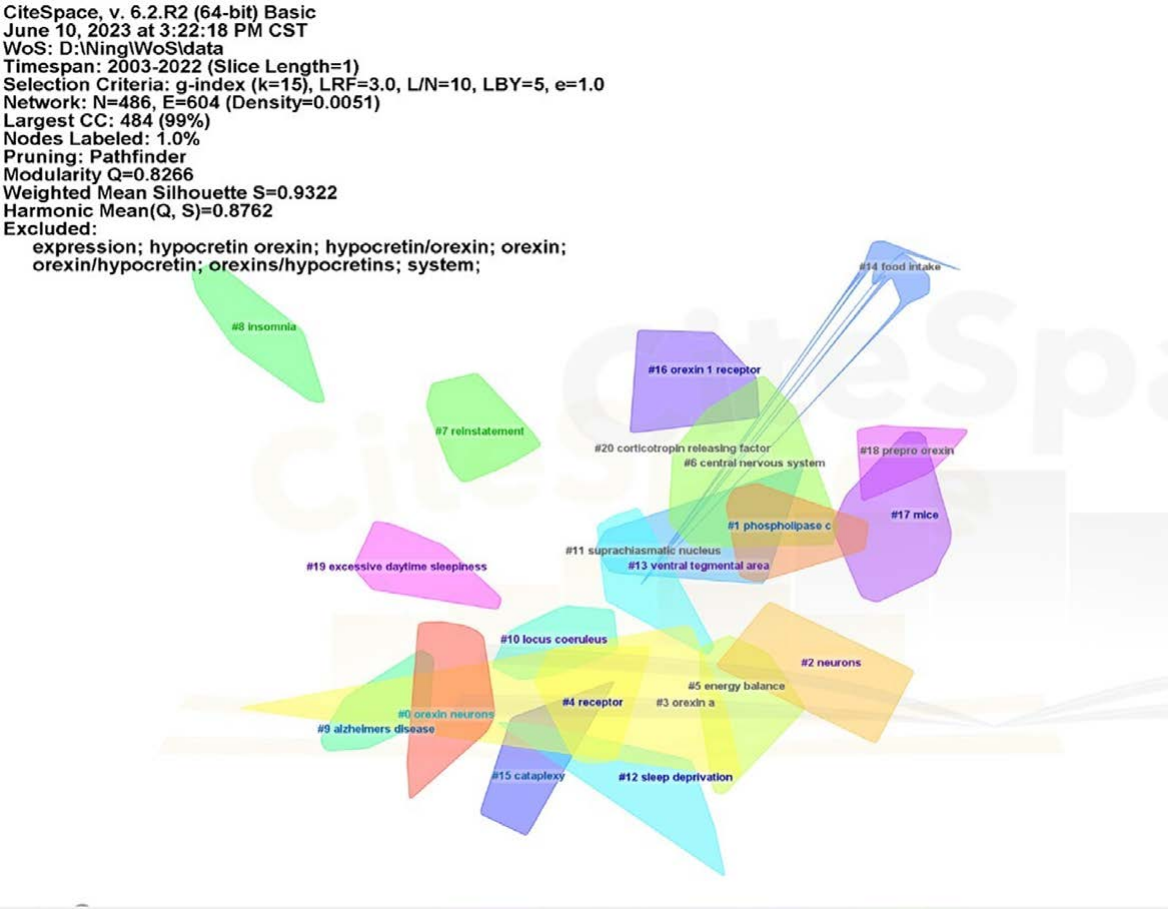
Clustered networks of keywords drawn via CiteSpace. The top 19 largest clusters are shown.

Finally, the keywords were divided into 19 different clusters to identify research hotspots in orexin studies (Fig. 9). Based on network structure and clarity of clusters, CiteSpace provided modularity (*Q* values) and silhouette values (*S* values).In general, *Q* values range from 0 to 1, with values >0.3 indicating significant community results. If *S* values exceed 0.5, the cluster is considered reasonable, while values above 0.7 indicate a convincing cluster. In this study, all clusters have *Q* values significantly >0.3 and *S* values exceeding 0.7, indicating robust clustering outcomes. The top 5 clusters are listed in Table 4.

**Table 4:** The top 5 clusters of keywords.

Cluster ID	Size	Silhouette	Mean (Year)	Keywords	Label (LSI)
0	34	0.949	2010	Orexin neurons	Sleep; hypothalamus; narcolepsy; orexin/hypocretin neurons; arousal lateral hypothalamus; food reward; heroin; dopamine transporter; slow-wave activity
1	33	0.939	2009	Phospholipase C	Orexin receptors; daily rhythm; sex difference; peripheral tissue; cannabinoid receptors ventral tegmental area; nucleus accumbens; d2 dopamine receptor; d1 dopamine receptor; plasticity
2	29	0.887	2008	neurons	Sleep; narcolepsy; hypocretin orexin; mutation; gene orexin; hypocretin; wakefulness; immunoreactivity; brainstem
3	28	0.916	2007	Orexin A	Blood pressure; rostral ventrolateral medulla; psychological stress; perifornical area; schlager mouse food intake; y1 receptor; paradoxical sleep; like-1 homolog; androgens
4	26	0.882	2008	Receptor lateral	Lateral hypothalamus; body weight regulation; dopaminergic receptor antagonists; single-unit activity; neural development orexin; habenula; circadian; per2; luciferase

Cluster 0: Relationship of orexins with sleep and metabolism.
Cluster 1: Mechanism and possible signaling pathways.
Cluster 2: Relationship between orexin and immunity.
Cluster 3: Association between orexin, hypertension, and mental stress.
Cluster 4: Relevance of orexins to neurodegenerative diseases.

## 4. Discussion

### 4.1. Distribution of annual publications

Over the last 20 years, from 2003 to 2022, our findings indicate that the number of publications on orexins has remained consistently substantial, underscoring the enduring significance of this research field. The productivity and development of a specific area can often be gauged by the volume of publications it generates, suggesting that orexin research continues to be a focal point for scholarly exploration. This sustained level of publication output implies that new scientific inquiries and challenges in orexin research continue to await further investigation by scholars in the field.

### 4.2. Analysis of countries or regions

As illustrated in Figure 4A, USA, Japan, and China are the countries with the highest publication volume in the field of orexin research. Figure 4B demonstrates that the USA and Japan collaborate more frequently with other countries, while China and Canada tend to conduct scientific research more independently.

### 4.3. Analysis of institutions

RLUK and Stanford University not only lead in publication volume in the field of orexin research but also maintain close connections with other institutions, making them significant contributors in this domain.

### 4.4. Analysis of authors

Yves Dauvilliers is the leading author in orexin research with 69 published studies, followed by Abbas Haghparast and Takeshi Sakurai. This analysis provides highly personalized scientific research information for other researchers. Yves Dauvilliers of Sleep and Wake Disorders Center in Paris, France, published his first paper in 1999, as indexed in the Web of Science. Since then, a total of 732 articles have been published in the Web of Science, garnering 1827 citations, with an impressive h-index of 71 and an average of 24.88 citations per article. The authors’ centrality values were all below 0.1, indicating limited collaboration among them, suggesting that enhancing communication among the authors is essential.

### 4.5. Analysis of keywords

#### 4.5.1. Milestones in orexin research: approval of double orexin receptor antagonists for clinical use

Orexins and their receptors (orexin/hypocretin receptors 1 and 2) are crucially involved in the regulation of sleep and wakefulness.^[16,17]^ Notably, orexin agonists and antagonists have been shown to effectively treat disorders such as excessive somnolence and insomnia. As shown in Figure 8, research on orexin receptor antagonists emerged as a hotspot in 2013. In 2014, suvorexant became the first dual orexin receptor antagonist approved by the FDA. Over the next nearly ten years, orexin receptor antagonists have remained popular, showing no signs of decline. It is reasonable to speculate that orexin receptor antagonists will remain a hot topic for several years. Current research is focusing on the impact of suvorexant on comorbid insomnia, while studies on lemborexant and daridorexant are directed towards primary insomnia.^[18–20]^ Despite progress, further evidence is necessary for the successful translation of almorexant into clinical applications for humans.

#### 4.5.2. Orexin neurons in the ventral tegmental area: essential regulators of reward processing and addiction behaviors

Opioid and alcohol addiction are characterized by compulsive drug-seeking behavior and withdrawal symptoms, with high rates of disability. These addictions are currently affecting tens of millions of people worldwide. Research suggests that the orexin system holds promise as a potential target for treating substance addiction.^[21]^ The orexin system regulates reward mechanisms and drug addiction through the involvement of multiple systems, including protein kinase C and dopamine, as well as various brain regions such as the lateral hypothalamus, perifornical area of the posterior thalamus, prefrontal cortex, and amygdala. In individuals with opioid abuse, increased levels of orexin-A have been detected. In rodent studies, injecting orexin into the brain can restore their preference for morphine. Conversely, in people who have quit methamphetamine, orexin-A levels in the urine remain low for an extended period. This may be due to the orexin system regulating the plasticity of protein kinase C alpha and glutamatergic synapses, which in turn affects the reward pathways. Some studies also suggest that the orexin system exerts its effects by interfering with anti-reward mechanisms. Multiple studies have shown that orexin receptor antagonists may be a potential approach for treating addiction. These antagonists can reduce opioid withdrawal symptoms, such as tremors and altered olfactory perception, and improve behaviors related to opioid and alcohol seeking.^[22–24]^

#### 4.5.3. Narcolepsy: the outcome of genetic and environmental mechanisms—orexin as a key factor

Narcolepsy is a brain disorder characterized by dysfunction or deficiency of orexin neurons in the lateral hypothalamus. Clinically, it manifests through symptoms such as cataplexy, excessive daytime sleepiness, and disrupted sleep–wake cycles. The onset of narcolepsy is believed to arise from the interplay of genetic and environmental factors, leading to an immune-mediated selective loss or dysfunction of orexinergic neurons.^[25]^ Consequently, individuals with narcolepsy exhibit elevated levels of tumor necrosis factor alpha and interferon-gamma in their serum, alongside activation of CD4+ and CD8+ T cells. Some individuals experience symptom improvement following immunotherapy. Importantly, narcolepsy is strongly associated with the human leukocyte antigen class II region, which encodes molecules responsible for presenting antigenic peptides to T cells.^[25,26]^ Notably, the presence of HLA-DQB1*06:02 indicates a potentially heightened risk for developing narcolepsy. Additionally, the T Cell Receptor Alpha Constant gene, involved in T-cell receptor α-constant, has been linked to narcolepsy, reinforcing the understanding that it is fundamentally an immune response-mediated condition.^[27–29]^ A phase II trial of the orexin 2 receptor agonist for treating narcolepsy type 1 has been completed. The orexin receptor 2 agonist led to greater improvements in measures of sleepiness and cataplexy compared to placebo over an 8-week period, although it was associated with hepatotoxic effects.

#### 4.5.4. Orexin receptor 1: a pivotal modulator of hypertension and stress responses in the hypothalamus

Increasing the levels of orexin receptor 1 in the neurons of the hypothalamic paraventricular nucleus enhances neuronal firing activity and sympathetic nerve activity, thereby exacerbating hypertension in obese Zucker Diabetic Fatty rats, a model used to study obesity-related hypertension. Conversely, blocking the orexin receptor through pharmacological intervention results in a reduction of blood pressure in spontaneously hypertensive rats. his finding underscores the role of the orexin system in salt-sensitive hypertension.^[30,31]^ Orexins play a crucial role in regulating the neurobiological systems that respond to stressful stimuli, with alterations in their levels observed in stress-related psychiatric disorders such as major depressive disorder and anxiety disorders.^[13,32]^ Consequently, orexins present themselves as potential targets for the treatment of these disorders.^[33]^

#### 4.5.5. Orexin receptor 2: is it involved in Alzheimer disease?

This suggests a potential relationship between orexins and neurodegenerative diseases. Orexinergic transmission, a crucial pathway in the body, can significantly affect the function of the hypothalamic suprachiasmatic nucleus, thereby influencing the regulation of circadian rhythms.^[34]^ Studies have revealed that orexins can modulate the clock cycle in the hippocampus through their input signals, leading to the precise regulation of clock gene expression, which may be linked to susceptibility to Alzheimer disease.^[35,36]^ Furthermore, orexinergic signaling plays a pivotal role in regulating tau protein-induced degeneration and the amplification of beta-amyloid levels, both of which are known pathological markers of Alzheimer disease.^[37,38]^ Notably, emerging research suggests that the administration of orexin receptor antagonists may hold promise in managing Alzheimer disease, offering potential therapeutic benefits for affected individuals.^[39,40]^ Selective orexin-2 receptor antagonists are currently undergoing clinical trials. Furthermore, dysfunction of the orexinergic pathway is implicated in the pathogenesis of Alzheimer disease, multiple sclerosis, and Huntington disease, and is associated with sleep impairment, pathogenic protein aggregation, neuronal loss, and the activation of neuroinflammation (Figs. 1 and 2). However, the exact roles of the orexinergic system in these diseases and the underlying mechanisms are still not fully understood. Therefore, it is essential to further investigate the dysregulation of the orexinergic system in neurodegenerative diseases and its role in their pathogenesis.

#### 4.5.6. Strengths and limitations

This literature analysis, based on visualization, can guide scholars in quickly understanding the research focus and development trends of orexin in neuroscience. To our knowledge, this is the first bibliometric analysis of orexin. The visual analysis of the literature offers a means for researchers to comprehend the key research topics, hotspots, and development trends in the field of orexin. However, this study also has certain limitations that need to be addressed. First, this study only searched for literature in the WoSCC database. While it is one of the most authoritative tools for retrieving scientific and technological literature, it does not encompass all research related to orexin. Second, due to the limitations of the extraction period, literature from 2023 and 2024 has not been fully included. Third, CiteSpace cannot fully replace systematic retrieval methods.

## 5. Conclusions

Orexin, a neuropeptide involved in various physiological and pathological processes, is associated with sleep/wakefulness, reward mechanisms, stress responses, and neurodegenerative diseases. It holds significant research value and potential applications in medical science. According to the visual analysis results from CiteSpace software, research on orexin is rapidly expanding. An increasing number of articles published in international core journals indicates a significant impact. The USA and Japan are the leading countries in terms of orexin research. However, the lack of collaboration among researchers suggests that enhancing academic exchange and cooperation among authors could further advance the field of orexin research.

## Author contributions

**Data curation:** Taiwei Lou, Zirong Li, Xiaowei Nie.

**Visualization:** Ming Zhao.

**Writing – original draft:** Ning Sun, Rui Wei, Bochao Jia.

**Writing – review & editing:** Wenxiao Yu, Ming Zhao, Qiuyan Li.
